# Evaluation of the Sphingolipidomic Profile in Women with Anorexia Nervosa: Relationships with Parameters Related to Body Composition, Cardiovascular Function, Glucometabolic Homeostasis, and Lipoprotein Metabolism

**DOI:** 10.3390/jcm14186482

**Published:** 2025-09-15

**Authors:** Antonello E. Rigamonti, Rita Paroni, Aldijana Sadikovic, Camillo Morano, Adele Bondesan, Diana Caroli, Francesca Frigerio, Laura Abbruzzese, Sandra Savino, Silvano G. Cella, Alessandro Sartorio

**Affiliations:** 1 Department of Clinical Sciences and Community Health, Dipartimento di Eccellenza 2023–2027, University of Milan, 20129 Milan, Italy; silvano.cella@unimi.it; 2 Department of Health Sciences, University of Milan, 20142 Milan, Italy; rita.paroni@unimi.it (R.P.); aldijana.sadikovic@unimi.it (A.S.); camillo.morano@unimi.it (C.M.); 3 Experimental Laboratory for Auxo-Endocrinological Research, Istituto Auxologico Italiano, Istituto di Ricovero e Cura a Carattere Scientifico (IRCCS), 28824 Piancavallo-Verbania, Italy; a.bondesan@auxologico.it (A.B.); d.caroli@auxologico.it (D.C.); f.frigerio@auxologico.it (F.F.); sartorio@auxologico.it (A.S.); 4 Division of Auxology, Istituto Auxologico Italiano, Istituto di Ricovero e Cura a Carattere Scientifico (IRCCS), 28824 Piancavallo-Verbania, Italy; l.abbruzzese@auxologico.it; 5 Division of Eating and Nutrition Disorders, Istituto Auxologico Italiano, Istituto di Ricovero e Cura a Carattere Scientifico (IRCCS), 28824 Piancavallo-Verbania, Italy; s.savino@auxologico.it

**Keywords:** anorexia nervosa, sphingolipids, body composition, cardiovascular function, glucometabolic homeostasis, lipoprotein metabolism, normal-weight healthy subjects

## Abstract

**Background.** Anorexia nervosa (AN) is a metabolic-psychiatric disease, in which, besides an extremely low body weight, there is a paradoxical dyslipidemia. **Objectives.** The aims of the present study were (1) to carry out a sphingolipidomic profiling in a group of women with AN (n = 28; body mass index [BMI]: 15.54 [13.96–16.32] kg/m^2^) compared to a normal-weight healthy (NWH) group (n = 30; BMI: 22.05 [19.67–23.72] kg/m^2^), and (2) to correlate plasma levels of single or total sphingolipids with parameters related to body composition, cardiovascular function, glucometabolic homeostasis, and lipoprotein metabolism. **Results.** Age, weight, BMI, fat mass (FM) (%), and fat-free mass (FFM) (kg) were significantly lower in women with AN than in the NWH group. Women with AN exhibited lower values of both systolic and diastolic blood pressure than the NWH group. Glucose, insulin, and HOMA-IR were significantly lower in women with AN than in the NWH group. Finally, C-reactive protein (CRP) was significantly higher in NWH women than in those with AN. Among all the sphingolipid species evaluated, 16 were significantly increased in AN (single species or whole family of summed species), while 6 were decreased. The highest significant correlations of sphingolipidomic profiling were mainly concentrated within parameters related to body composition. **Conclusions.** Women with AN have a specific sphingolipidomic profile that could hopefully serve as a marker for monitoring treatment effectiveness.

## 1. Introduction

Anorexia nervosa (AN) is a metabolic-psychiatric disease [1,2], characterized by a high mortality rate [3,4,5,6,7]. In this respect, despite relevant progress in psychiatric, endocrinological, and nutritional research in the last decade, there is no evidence for a definitively “effective” treatment [1], so that about 30% of patients complain about long-term chronicizing and/or relapsing patterns of the disease [7,8,9,10], with a variability in percentages, due to out- or inpatient follow-up and duration of the follow-up.

Although the pathophysiological role of genetics in AN is increasingly becoming evident [2], genetics incompletely explains the pathophysiology of AN, and other psycho-biological mechanisms underlying AN might be involved.

For a long time, clinical endocrinology has defined the existence of a “metabolic” component in AN, but only recently, studies in the field of biological psychiatry have demonstrated the association between metabolic vs. psychiatric components in AN. In this context, genome-wide studies have identified a series of gene *loci* that are referred to as the endocrine-metabolic system (metabolic component) and neurobiology (psychiatric component) [2,11]. Specifically, a positive association between AN and high-density lipoprotein cholesterol (HDL-C) has been recognized [2,11]. Moreover, a meta-analysis has evidenced that a biochemical characteristic in AN is a co-existing condition of dyslipidemia, paradoxically similar to that found in obesity and metabolic syndrome [12]. Nevertheless, AN-related dyslipidemia, which might be biochemically a phenotypical trait of the disease, but also a statistically heterogeneous biochemical surrogate, depends on a vast number of confounding factors, including, in the context of AN, nutritional therapy, partial body weight gain, age (adolescent vs. adult women), duration within a normal body weight (short vs. long time), body mass index (BMI), and body composition (pre- vs. post-admission to hospital or discharge vs. follow-up), and glucometabolic homeostasis [12]. Therefore, there is a pressing need for further clinical studies that consider the impact of these confounding factors on AN-related dyslipidemia before investigating the pathophysiological link between dyslipidemia and AN.

Lipidomics, including sphingolipidomics, is emerging as a valuable analytical methodology, applicable on a large scale, for studying a vast number of lipid classes and single lipid compounds, which act as metabolites of biochemical pathways within the cell or subcellular structures [13]. In this context, lipids serve for the assembly and function of the plasma membrane, energy storage, and the regulation of signalling pathways (ligands of receptors or modulators of enzymes) [14,15]. In particular, some lipids, such as sphingolipids, are considered “bioactive” [16].

Parallelly, dyslipidemia, defined in clinical practice as “generic” alteration of lipid metabolism, has been associated with a series of diseases, including atherosclerosis, diabetes mellitus, obesity, chronic inflammation, and cancer [17], besides some neurological and psychiatric diseases [18]. Surprisingly, total cholesterol (T-C), a simple biochemical analyte, has been positively associated with suicidality in AN [19].

The lipidomics, including sphingolipidomics, possesses, when compared to standard clinical biochemistry, the potential of characterizing the lipid and, specifically, sphingolipid profile in a patient with AN [20]. So, rather than a “generic” (and also gross) AN-related dyslipidemia (e.g., high plasma levels of T-C and other standard lipids), it is possible, not only in an experimental setting, but also in the clinical practice, to associate plasma levels of a vast number of lipids (or sphingolipids) with demographic, biochemical, and clinical parameters of the disease.

Recently, in a population of female adolescents with AN, Tam et al. [20] have carried out a lipidomic investigation in the acute phase of the disease and after a partial body weight gain, attained through a short-term program of metabolic rehabilitation. Before hospital admission, patients with AN exhibited high plasma levels of lipids from different classes compared to those in a control group [20]. Unfortunately, in this study, only a limited number of sphingolipids (in terms of classes and compounds) have been determined. Similar results have been obtained in the study by Hussain et al. [21].

Thus, based on the previous considerations, the primary endpoint of the present study was to determine the sphingolipidomic profile in women with AN compared to a normal-weight healthy (NWH) group. Secondarily, given the existence of different sphingolipidomic profiles between AN and NWH groups, we evaluated associations of the sphingolipid profile with demographic, biochemical, and clinical parameters.

## 2. Materials and Methods

### 2.1. General Information

A cross-sectional design was employed in the present study to determine plasma sphingolipidomics in AN and to assess the associations between plasma levels of individual sphingolipids (or sphingolipid classes) and demographic, biochemical, and clinical parameters.

### 2.2. Subjects

The present study included 28 women with AN: 25 patients were amenorrheic, 1 had normal menses, and 2 were under oral contraceptive therapy. Participants were consecutively recruited at the Division of Eating and Nutrition Disorders at Istituto Auxologico Italiano, IRCCS, Piancavallo-Verbania, a tertiary care centre specialised in the multidisciplinary (metabolic, nutritional, and psychological) rehabilitation of severe obesity and eating disorders in Northern Italy.

Participants were eligible for the study if they were female (due to the higher prevalence of eating disorders in women compared to men [22]), between 18 and 60 years old, and had a diagnosis of AN based on the Diagnostic and Statistical Manual of Mental Disorders, Fifth Edition (DSM-5), as determined by the Structured Clinical Interview (Clinical Version 5—SCID-5 CV) [23]. All patients provided written informed consent before participation. Women were excluded if they had a comorbid psychiatric disorder diagnosed according to DSM-5 criteria or any other medical condition that might affect their ability to participate in the study.

Participants included in the study were at their first admission to our centre, with a previous history of only outpatient visits.

Patients with AN underwent a multidisciplinary inpatient rehabilitation treatment in a residential setting for three to eight weeks, as previously described [24]. The treatment included both individual and in-group sessions, focusing on the bodily experience. Body image difficulties were treated through BI therapy [25]. Low-intensity adapted physical activities were proposed to meet the patients’ desire for movement [26,27,28], targeting it as part of a healthy lifestyle. Psychological functioning (e.g., depression, anxiety, and post-traumatic symptomatology) was targeted through both individual and in-group psychotherapy [29]. Finally, individual and in-group consultations with nutrition experts were delivered to identify tailored dietary schemes and to promote nutritional education about biological hunger, satiety, and nutrients [30]. Participants were monitored during all the meals by dietitians and nurses (assisted meal).

NWH female controls (n = 30) were recruited from the hospital’s medical, research, and administrative staff, as well as among friends and colleagues. The control group excluded persons with representative disorders, such as psychiatric disorders. Convenience sampling was utilized due to its practicality in accessing readily available populations. Although this approach inherently limits generalizability, efforts were, however, made to mitigate these limitations by targeting subjects of the same educational and social status.

The study was approved by the Ethical Committee no. 5, Lombardy Region (registration number: 209/24; date of approval: 23 April 2024; research order code: 01C417, acronym: SFINGOANNER). All procedures were conducted in accordance with the Helsinki Declaration of 1975 and its subsequent amendments, or with comparable ethical standards.

### 2.3. Anthropometric Measurements

A scale with a stadiometer was used to determine height and weight (Wunder Sa.Bi., WU150, Trezzo sull’Adda, Italy). Body composition was measured by bioimpedance analysis (Human-IM Scan, DS-Medigroup, Milan, Italy) after 20 min of supine rest. BMI, fat mass (FM), and fat-free mass (FFM) were determined in all subjects.

### 2.4. REE Measurement

Resting energy expenditure (REE) was measured between 8:00 and 10:00 a.m. in thermoneutral conditions (room temperature: 22–25 °C) using an open-circuit, indirect, computerized calorimeter equipped with a canopy (Vmax 29, Sensor Medics, Yorba Linda, CA, USA). The calorimeter underwent periodic quality control tests to ensure the reliability of the measurements. The gas analyzers were calibrated before each test using a reference gas mixture of 15% O_2_ and 5% CO_2_. The participants were fasting for at least 8 h, had not smoked for at least 1 h, and waited 30 min in a sitting position before undergoing REE measurement. REE was assessed in the supine position for at least 30 min, including an acclimation period of 10 min. The data related to the acclimation period were discarded. The steady state was defined as at least 5 min with less than 5% variation in the respiratory quotient and ventilation [31]. After the steady state was reached, O_2_ consumption and CO_2_ production were recorded at 1 min intervals for at least 20 min and averaged over the entire measurement period. REE was calculated from O_2_ consumption and CO_2_ production using Weir’s equation [32].

### 2.5. Blood Pressure and Heart Rate

Blood pressure was measured on the right arm using a sphygmomanometer with an appropriately sized cuff, with the subject in a seated position and in a relaxed condition. The procedure was repeated three times at 10 min intervals; the means of the three values for systolic blood pressure (SBP) and diastolic blood pressure (DBP) were recorded.

Heat rate (HR) was measured using both manual and electronic methods on two separate occasions.

### 2.6. Metabolic Variables

Blood samples (about 10 mL) were collected at around 8:00 a.m. after an overnight fast (about 12 h) on the third day post-admission to the hospital, before starting the intervention of metabolic rehabilitation.

Total cholesterol (T-C), high-density lipoprotein cholesterol (HDL-C), low-density lipoprotein cholesterol (LDL-C), triglycerides (TG), glucose, insulin, and C-reactive protein (CRP) were measured.

Colorimetric enzymatic assays (Roche Diagnostics, Monza, Italy) were used to determine serum T-C, LDL-C, HDL-C, and TG levels. The sensitivities of the assays were 3.86 mg/dL (1 mg/dL = 0.03 mmol/L), 3.87 mg/dL (1 mg/dL = 0.03 mmol/L), 3.09 mg/dL (1 mg/dL = 0.03 mmol/L), and 8.85 mg/dL (1 mg/dL = 0.01 mmol/L), respectively.

Serum glucose levels were measured using the glucose oxidase enzymatic method (Roche Diagnostics, Monza, Italy). The sensitivity of the method was 2 mg/dL (1 mg/dL = 0.06 mmol/L).

Serum insulin concentration was determined using a chemiluminescent immunoassay with a commercial kit (Elecsys Insulin, Roche Diagnostics, Monza, Italy). The sensitivity of the method was 0.2 µIU/mL (1 µU/mL = 7.18 pmol/L).

CRP was measured using an immunoturbidimetric assay (CRP RX, Roche Diagnostics GmbH, Mannheim, Germany). The sensitivity of the method was 0.03 mg/dL.

The intra- and inter-assay coefficients of variation (CVs) were the following: 1.1% and 1.6% for T-C, 1.2% and 2.5% for LDL-C, 1.8% and 2.2% for HDL-C, 1.1% and 2.0% for TG, 1.0% and 1.3% for glucose, and 1.5% and 4.9% for insulin.

For each patient, we also calculated the homeostatic model assessment of insulin resistance (HOMA-IR) according to the following formula: (insulin (μIU/mL] × glucose (mmol/L))/22.5.

### 2.7. Lipid Extraction from Plasma and Sphingolipid Target Analysis by LC-MS/MS

Sphingolipids extraction and targeted LC–MS/MS analysis were performed as previously described [33,34,35]. Plasma (25 µL) was added with the internal standard mixture (10 µL, Cer 12:0, SM 12:0, GluCer 12:0, and Sph d17:0, 20 µM), diluted with water (75 µL), and mixed with a methanol/chloroform solution (850 µL, 2:1, *v*/*v*). The lipids were extracted by ice-sonication (30 min) and then subjected to thermo-shaking (1 h, 1000 rpm, 38 °C). Then, they went through alkaline methanolysis (75 µL KOH 1 M in methanol, thermo-shaking 2 h at 38 °C) and were neutralized by the addition of 75 µL 1 M acetic acid in MeOH. The organic phase was separated via centrifugation (25 min at 13,400 rpm), and 950 µL was transferred and evaporated in a SpeedVac vacuum concentrator. The residuals were dissolved in 150 µL of methanol + 0.5 mM BHT, centrifuged again for 10 min at 13,400 rpm, withdrawn into a glass vial, and 10 µL of clear supernatant was directly injected into the LC-MS/MS instrument for quantitative analysis. If the samples were cloudy, the tip-tap filtration method, as described earlier [35], was applied. Samples were analyzed by LC Dionex 3000 UltiMate (ThermoFisher Scientific, Waltham, MA, USA) coupled to a tandem mass spectrometer AB Sciex 3200 QTRAP (AB Sciex, Framingham, MA, USA) equipped with an electrospray ionization TurboIonSpray™ source operating in positive mode (ESI+). The separation was achieved either using a reverse-phase Acquity BEH C8 column 1.7 µm, 2.1 × 100 mm (Waters, Milford, MA, USA) (for ceramides, dihydroceramides, and sphingomyelins) or a reversed-phase Cortecs C18 1.6 µm, 2.1 × 100 mm (Waters, Milford, MA, USA) column (for sphingoid bases) by mixing eluent A (0.2% formic acid 2 mM ammonium formate water solution) and eluent B (methanol 0.2% formic acid 1 mM ammonium formate). The lipidomic target analysis comprised forty-three sphingolipid species, the most representative ceramides, dihydroceramides, sphingomielins, hexosil, lactosil, GM3 species, plus four sphingoid bases (sphingosine, dihydrosphingosine, sphingosine-1P, and dihydrosphingosine-1P); for the abbreviations, see the list below-reported. A six-point calibration curve for each analyte was evaluated by spiking increasing amounts of the analytes in water, covering a concentration range of 0–40 pmol/vial. Linearity was observed for each compound in the whole range (R^2^ > 0.99). Quantitative analysis was performed by interpolating each peak area of the analyte/area IS with the calibration curve slope for each sphingolipid. The sphingolipid amount was expressed in µmol/L.

### 2.8. Statistics

Sigma Stat 4.0 (SysStat Software Inc., Palo Alto, CA, USA) and GraphPad PRISM 7.0a (La Jolla, CA, USA) were used for analyses and plotting.

Parameters were expressed as median (interquartile range) and analyzed by Mann–Whitney Rank Sum Test (AN vs. NWH). Categorical variables were compared through chi-square or Fisher tests.

Spearman’s correlation was used to calculate the correlation of single sphingolipids or sphingolipid classes with demographic, biochemical, and clinical parameters. A heat map representation was used to show, in a colour-coded system, the correlation coefficients and the corresponding *p*-values (yes or no).

A *p*-value < 0.05 was considered statistically significant.

## 3. Results

### 3.1. Demographic, Biochemical, and Clinical Parameters

Table 1 reports comparisons of demographic, biochemical, and clinical parameters between the AN and NWH groups.

In brief, age, weight, BMI, FFM (kg), and FM (%) were significantly lower in women with AN compared to the NWH group. Despite the similar HR, women with AN exhibited lower values of both SBP and DBP than the NWH group. When considering the glucometabolic homeostasis, glucose, insulin, and HOMA-IR were significantly lower in women with AN than in the NWH group, with similar values for HbA1_c_. No significant differences were found in T-C, LDL, HDL, and TG between the AN and NWH groups. Finally, CRP was significantly higher in NWH women than in those with AN.

### 3.2. Sphingolipidomic Profile

Table 2 reports comparisons of single sphingolipids and sphingolipid classes between the AN and NWH groups.

In total, among single sphingolipidis and sphingolipid classes, 22 comparisons between the AN and NWH groups were significant. In particular, plasma levels of Cer 20:0, Cer 24:1, DHCer 24:1, SM 16:0, SM 24:0, SM 24:1, total SM, HexCer 24:1, LacCer 18:1, LacCer 24:0, LacCer 24:1, GM3 16:0, GM3 20:0, GM3 24:0, GM3 24:1, and DHS1P were significantly higher in women with AN than NWH group; on the contrary, plasma levels of Cer 22:0, DHCer 18:1, LacCer 18:0, GM3 18:1, total LacCer, and DHSph were significantly lower in women with AN than NWH group.

### 3.3. Correlations of Sphingolipids with Other Parameters

Demographic, biochemical, and clinical parameters were subdivided into four macro-groups related to the following: body composition; cardiovascular function; glucometabolic homeostasis; and (standard) lipid profiles. Single sphingolipids and sphingolipid classes, which were significantly different when comparing AN and NWH groups (see above), were correlated with these parameters. The results are summarized in Tables S1–S4, included in the Supplementary Material.

**Figure 1:**
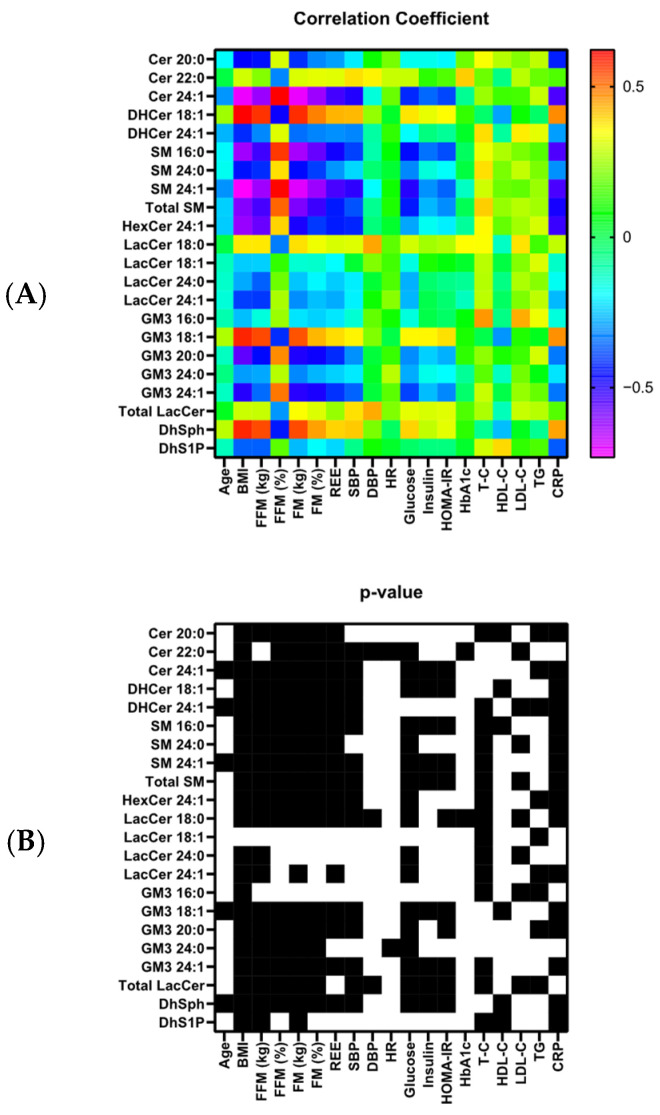
Correlations of the twenty-two (single or total) sphingolipids that were found to be significantly different when comparing AN vs. NWH groups (see Table 2). Heat maps display correlation coefficients (panel (**A**)—(**above**)) and significance (panel (**B**)—(**below**)). Please note that the significance was represented by black (significant correlation) or white (non-significant correlation).

To facilitate the interpretation of correlations, panels A and B of Figure 1 display heat maps that report the coefficients and their corresponding significance levels. The highest significant correlations of sphingolipidomic profiling were mainly concentrated within parameters related to body composition.

## 4. Discussion

In the present study, carried out in a group of young women with AN, hospitalized for a multidisciplinary program of metabolic rehabilitation, compared to an NWH group, a plasma sphingolipidomic approach was adopted, and twenty-two sphingolipid compounds and classes were found to be different between AN and NWH groups: in particular, plasma levels of Cer 20:0, Cer 24:1, DHCer 24:1, SM 16:0, SM 24:0, SM 24:1, total SM, HexCer 24:1, LacCer 18:1, LacCer 24:0, LacCer 24:1, GM3 16:0, GM3 20:0, GM3 24:0, GM3 24:1, and DHS1P were higher in women with AN than NWH group; on the contrary, plasma levels of Cer 22:0, DHCer 18:1, LacCer 18:0, GM3 18:1, total LacCer, and DHSph were lower in women with AN than NWH group. Furthermore, the sphingolipidomic profile was correlated with demographic, biochemical, and clinical parameters, which were clustered in four groups: parameters related to body composition, cardiovascular function, glucometabolic homeostasis, and (standard) lipid metabolism. The statistically different sphingolipid compounds and classes, as listed above, were mainly correlated with parameters related to body composition.

Body composition, specifically BMI, FM, and FFM, is recognized to influence sphingolipid metabolism and circulating levels. Increased BMI and FM, often associated with obesity and metabolic syndrome, are linked to elevated levels of some sphingolipids, particularly Cers, both in adipose tissue and plasma, potentially contributing to high cardiometabolic risk in these patients [36]. Conversely, increased FFM has been associated with other sphingolipids, particularly increased plasma levels of SMs, endowed with beneficial metabolic properties, including an improvement in insulin sensitivity [37].

At the molecular level, sphingolipids play crucial roles in cell signalling, membrane structure, and inflammation, all of which are influenced by body composition [14,15,16].

For instance, fatty acids, released from adipose tissue due to activation of lipolysis, are needed to synthesize Cers and other sphingolipids via the so-called de novo pathway (see below). Moreover, inflammation associated with obesity has also been demonstrated to stimulate sphingolipid synthesis [38].

Different from obesity, AN in females is characterized by fat loss in absolute terms, with fat redistribution, including a preferred decrease in extremity fat (i.e., subcutaneous white adipose tissue), and a paradoxical increase in trunk fat (i.e., visceral white adipose tissue) in percentage terms [39,40]. When considering brown adipose tissue, there is evidence of a reduced anatomic amount and/or functional activity in AN [41].

In the present study, though body composition, particularly FM, was grossly measured by the bioimpedance method without discriminating visceral vs. subcutaneous compartments, FM was dramatically reduced in women with AN compared to the NWH group. As adipose tissue represents one of the most relevant sources of synthesis and release of sphingolipids into the plasma [42], the differences in sphingolipidomic profiles that were found in the present study, between AN and NWH groups, might depend on an altered body composition, mainly FM. Alternatively (or additionally), as adipose tissue also represents a storage site where sphingolipids derived from other organs, such as the liver, are accumulated, the increased plasma levels of several sphingolipids in our women with AN (precisely, 16 to 22 vs. the NWH group) might be the consequence of the spill-over phenomenon due to the limited fat compartment in AN [43].

Similar considerations may be extended to muscle tissue, another organ involved in the synthesis, release, and storage of sphingolipids [44], which is typically reduced in starvation conditions, such as AN, due to stimulation of proteolysis, as demonstrated in the present study, with lower FFM in women with AN compared to the NWH group [45].

If fat redistribution and muscle shrinkage are mainly due to the underlying eating disorder (e.g., extremely limited energy intake and ensuing starvation) [40], the endocrine disruption that characterizes AN is supposed to be the most important mechanism underlying the peculiar metabolism of sphingolipids and sphingolipidomic profile in AN [39].

For instance, AN is fundamentally characterized by high plasma levels of ghrelin and growth hormone (GH), together with GH resistance, normal–low insulin-like growth factor 1 (IGF-1), and hypoinsulinemia [46,47,48,49,50,51]. Although the physiological effects of ghrelin, GH, and IGF-1, which are hormones affecting lipid metabolism, on sphingolipids have not been fully investigated, the different sphingolipidomic profile in AN, as identified in the present study, is supposed to be a consequence of the complex interplay among ghrelin, GH, IGF-1, and insulin, which is, as described above, disrupted in AN [39].

AN-related endocrine disruption encompasses other hormones, which regulate lipid and, presumably, sphingolipid metabolism.

For example, circulating levels of leptin, an adipocyte-derived anorexigenic peptide, are reduced in AN [52]. The regulatory role of leptin on glucose and lipid metabolism is direct through specific receptors expressed in many tissues [53] but also indirect through inhibition of kisspeptin, a hypothalamic peptide involved in the regulation of pulsatile gonadotropin-releasing hormone (GnRH) secretion [54]. The AN-related hypoestrogenism contributes to fat redistribution, lipid metabolism, and dyslipidemia [55]. This might also impact the sphingolipidomic profile in AN. Further studies are mandatory to investigate the relationship between estrogens and plasma sphingolipidomic profiling [56,57,58].

Under conditions of starvation, such as AN, increased corticotropin-releasing hormone (CRH) hypothalamic expression promotes the release of adrenocorticotropic hormone (ACTH) from the anterior pituitary [59]. ACTH then targets the adrenal cortex to stimulate the production of cortisol, which, in adipocytes, facilitates lipid accumulation in the presence of insulin, while promoting lipid mobilization in the presence of GH [60]. Taking into account the role of glucocorticoids in glucose and lipid metabolism, the disregulation of the hypothalamic–pituitary–adrenal (HPA) axis in AN might represent another mechanism underlying the different sphingolipidomic profiles that were identified, in the present study, between AN and NWH groups.

Notably, elevated cortisol suppresses the release of thyroid-stimulating hormone (TSH) from the anterior pituitary [61], which is also inhibited by ghrelin [62]. Hypothyroidism is often associated with AN [55], and hypothyroidism-related dyslipidemia might include sphingolipid metabolism, a topic that should be further investigated in lipidology and sphingolipidology.

Typically, AN is defined as chronic starvation without inflammation. Patients with AN appear to have lower CRP levels [63], a finding that was also observed in the present study. In any case, in AN, plasma concentrations of TNF-α, a proinflammatory cytokine, are higher than those in a control group [64]. TNF-α is known to alter plasma levels of several sphingolipids, particularly Cers and Sph, by activating sphingomyelinases, which break down SMs into Cers and Sph [65]. This process can be, at least in part, envisaged in the present study by considering the increased levels of some Cers in AN. Furthermore, plasma levels of some sphingolipids were correlated with CRP.

Although body composition is strictly related to glucometabolic homeostasis, herein, we have focused on the relationship between body composition and sphingolipids. Our results reveal several correlations between specific sphingolipids and parameters related to glucometabolic homeostasis, including glucose, insulin, and HOMA-IR. Disruption of glucometabolic homeostasis in AN is considered a counter-regulatory response to the condition of starvation, characterized by dramatic changes in body composition, such as BMI [53]. So, we argue that the peculiar sphingolipidomic profile in AN that was identified in the present study is only “indirectly” associated with glucometabolic homeostasis, being weight loss the *primum movens* that results in alteration of body composition, which, in turn, leads to alteration of glucometabolic homeostasis [66].

Similar considerations apply to interpreting the correlations we found between certain sphingolipids and parameters related to cardiovascular function [67].

Some Authors have postulated that the lipidomic profile in AN, which is similar to that in obesity and metabolic syndrome, is the deleterious consequence of the refeeding, which represents a part of the rehabilitative program administered to hospitalized patients with AN [20]. Unlike other studies, the present study did not observe dyslipidemia, as plasma levels of T-C, LDL-C, and TG were similar between AN and NWH. This would be indicative that the dyslipidemic phase, which occurs early in refeeding, was missing when blood sampling was performed in our women with AN (i.e., 2 days before starting our rehabilitative intervention). To address this issue, sphingolipidomic profiling should be conducted at various time points during the clinical course of the disease.

The potential clinical implications of our results are significant: in fact, the herein identified sphingolipidomic profile (or even one specific sphingolipid) might serve as a biochemical marker for monitoring treatment effectiveness and/or achieving (if possible) recovery from the disease.

At the molecular level, the length of the acyl group in Cers is primarily determined by the substrate specificity of different ceramide synthase (CerS) enzymes. Mammals have six CerS enzymes (CerS1–6), each preferentially utilizing acyl-CoA molecules with specific fatty acid chain lengths [68]. For instance, CerS5 favours palmitoyl-CoA (16 carbons), while CerS2 prefers acyl-CoAs with longer chain fatty acids (22–24 carbons) [69]. The expression of these CerS enzymes also varies between tissues, contributing to the diversity of Cer species found in circulation.

In the present study, increased plasma levels of long-chain-fatty-acid-acyl-containing sphingolipids, precisely HexCer 24:0, LacCer 24:0, LacCer 24:1, GM3 24:0, GM3 24:1, SM 24:0, and SM 24:1, were observed with congruently increased plasma levels of Cer 24:1, which, as substrate, is used for the synthesis of the other so-reported sphingolipids. This biochemical pathway might be related to an increased availability of very long-chain fatty acids in AN. In this context, in a study, after a short-term rehabilitative treatment, plasma levels of very long chain fatty acids (i.e., C22:0 and C24:0) remained high in patients with AN relative to the control group, suggesting a potential biochemical marker to monitor the nutritional status in this disease [70].

Similarly, DHCer 24:1 (increased in AN) might be formed through the N-acylation of DHSph (decreased in AN) by CerS2 using acyl-CoAs of very long chain length, such as C24:1 (presumably, increased in AN) [68].

Before closing, some strengths and limitations of our manuscript should be mentioned.

Regarding the strengths, the topic is novel, as it involves sphingolipidomic profiling in the context of AN, which is rare. Our results, though preliminary, will contribute to understanding the interaction between lipid metabolism and the pathophysiology of AN. Furthermore, we employed an advanced methodology that enables the quantitative analysis of multiple sphingolipid species, thereby strengthening the reliability of our results.

Regarding limitations, the relatively small sample size might have limited statistical power, and this might impede us from generalizing results to the entire population.

Carrying out the study exclusively in women significantly limits the applicability of the results to the broader AN population. In this regard, contemporary epidemiological data indicate rising incidence rates among boys and men [6]. In the future, it would be worth considering research in male groups.

The lack of a prospective experimental design and longitudinal data prevents an assessment of the dynamics of sphingolipidomic changes during therapy or recovery. Indeed, our future intention is to reproduce the same protocol by adopting a prospective experimental design.

Finally, in the present study, body composition was evaluated using bioelectrical impedance, a methodology that does not permit the estimation of body distribution of adipose tissue, including the distinction between visceral and subcutaneous compartments. Nevertheless, our argumentation regarding the role of adipose tissue in sphingolipidomics in AN, supported by evidence from the biomedical literature [39,40], is helpful in tentatively interpreting our results.

## 5. Conclusions

When considering the female population, a specific sphingolipidomic profile characterizes AN when compared to the NWH group. AN-related body composition, particularly fat loss and redistribution, as well as the ensuing endocrine disruption, may be the mechanisms underlying alterations in sphingolipid metabolism. Though the relationship between dyslipidemia and AN needs further investigation, sphingolipidomics might represent a biochemical approach for monitoring therapeutic success and clinical status in AN.

## Figures and Tables

**Table 1:** Demographic, biochemical, and clinical characteristics of subjects recruited in the study, subdivided into two groups: subjects with anorexia nervosa (AN) and normal-weight healthy (NWH) subjects.

Parameter	AN	NWH	*p*
N	28	30	-
Age (year)	23.7 [19.8–30.9]	28.1 [26.0–32.1]	0.006
Height (cm)	1.63 [1.56–1.70]	1.67 [1.62–1.70]	0.112
Weight (kg)	39.3 [36.1–42.1]	59.5 [54.9–66.4]	<0.001
BMI (kg/m^2^)	15.5 [13.9–16.3]	22.0 [19.7–23.7]	<0.001
FFM (kg)	33.9 [32.7–38.2]	46.0 [41.7–49.2]	<0.001
FM (%)	11.3 [6.5–15.3]	22.0 [18.3–29.0]	<0.001
SBP (mmHg)	100 [90–108]	117 [110–120]	<0.001
DBP (mmHg)	60 [60–70]	70 [65–72]	0.019
HR (beats/min)	72.5 [64.0–86.7]	70.0 [68.0–73.0]	0.395
REE (kcal)	1222 [1189–1276]	1409 [1311–1559]	<0.001
Glucose (mg/dL)	77.5 [70.5–84.0]	86.5 [82.0–90.0]	<0.001
Insulin (mU/L)	3.4 [2.3–6.7]	7.0 [5.1–9.1]	<0.001
HOMA-IR	0.67 [0.42–1.24]	1.50 [1.10–1.80]	<0.001
Hb1A_c_ (%)	5.05 [4.60–5.20]	5.10 [4.90–5.30]	0.090
T-C (mg/dL)	188 [149–219]	173 [157–201]	0.479
HDL-C (mg/dL)	71.5 [63.2–84.7]	68.5 [62.7–76.5]	0.308
LDL-C (mg/dL)	106.5 [77.7–129.7]	103.5 [85.2–122.0]	0.895
TG (mg/dL)	79.0 [60.5–92.7]	64.0 [47.7–87.5]	0.290
CRP (mg/dL)	0.00 [0.00–0.00]	0.100 [0.01–0.20]	<0.001

Note: Data, expressed as median and interquartile range [25th and 75th], were analyzed by the Mann–Whitney Rank Sum Test. See the text for abbreviations.

**Table 2:** Plasma sphingolipidomics in the study population: subjects with anorexia nervosa (AN) and normal-weight healthy (NWH) subjects.

Sphingolipid	AN				NWH		*p*
(µmol/L)	Median	25th	75th	Median	25th	75th	
Single							
Cer 14:0	0.018	0.015	0.021	0.016	0.013	0.019	=0.100
Cer 16:0	0.511	0.455	0.589	0.471	0.421	0.516	=0.109
Cer 18:1	0.014	0.009	0.017	0.014	0.011	0.016	=0.403
Cer 18:0	0.091	0.062	0.119	0.078	0.066	0.107	=0.423
Cer 20:0	0.183	0.140	0.204	0.109	0.073	0.151	<0.001
Cer 22:0	0.523	0.378	0.608	0.616	0.457	0.688	=0.027
Cer 24:1	1.660	1.522	1.813	0.936	0.742	1.111	<0.001
Cer 24:0	4.670	3.302	5.676	3.891	3.291	5.149	0.498
DHCer 16:0	0.017	0.014	0.024	0.020	0.018	0.029	=0.105
DHCer 18:1	0.000	0.000	0.000	0.004	0.000	0.006	<0.001
DHCer 18:0	0.007	0.005	0.009	0.0060	0.004	0.008	=0.608
DHCer 24:1	0.142	0.125	0.165	0.078	0.060	0.111	<0.001
DHCer 24:0	0.161	0.118	0.206	0.179	0.151	0.237	=0.084
SM 16:0	199.394	181.426	210.668	131.678	118.543	164.700	<0.001
SM 18:0	48.599	34.101	56.528	39.373	32.842	48.384	=0.063
SM 18:1	27.544	21.628	32.918	24.177	20.809	29.556	=0.228
SM 24:0	74.601	65.881	88.859	37.160	13.185	67.927	<0.001
SM 24:1	167.730	142.939	185.807	62.379	34.449	101.294	<0.001
HexCer 16:0	1.435	1.212	1.692	1.496	1.225	1.710	=0.720
HexCer 18:0	0.258	0.204	0.344	0.264	0.213	0.341	=0.969
HexCer 18:1	0.006	0.0045	0.009	0.016	0.009	0.034	=1.000
HexCer 20:0	0.319	0.263	0.388	0.299	0.266	0.393	=1.000
HexCer 22:0	4.021	3.565	5.181	4.570	3.984	5.257	=0.120
HexCer 24:0	4.747	4.459	6.268	5.051	4.281	5.954	=0.715
HexCer 24:1	6.912	5.982	7.972	4.017	3.428	5.733	<0.001
LacCer 16:0	6.514	5.740	7.520	7.031	5.779	9.453	=0.253
LacCer 18:0	0.098	0.078	0.115	0.119	0.090	0.201	=0.032
LacCer 18:1	0.069	0.050	0.092	0.053	0.040	0.069	=0.018
LacCer 20:0	0.100	0.068	0.120	0.087	0.040	0.112	=0.164
LacCer 22:0	0.436	0.331	0.508	0.393	0.091	0.498	=0.213
LacCer 24:0	0.402	0.328	0.520	0.227	0.009	0.379	=0.001
LacCer 24:1	2.775	2.201	3.324	1.362	0.186	2.112	<0.001
GM3 16:0	2.460	2.183	2.947	1.864	1.610	2.594	=0.007
GM3 18:0	0.427	0.357	0.583	0.341	0.250	0.583	=0.088
GM3 18:1	0.000	0.000	0.000	0.024	0.000	0.024	<0.001
GM3 20:0	0.207	0.160	0.267	0.120	0.070	0.144	<0.001
GM3 22:0	0.660	0.570	0.950	0.793	0.547	1.387	=0.171
GM3 24:0	0.307	0.223	0.430	0.213	0.145	0.288	=0.008
GM3 24:1	1.453	1.103	2.203	0.781	0.593	1.191	<0.001
Sph	0.103	0.091	0.110	0.098	0.087	0.120	=0.963
S1P	2.448	1.610	3.311	1.866	1.314	3.726	=0.246
DHSph	0.000	0.000	0.000	0.013	0.000	0.016	<0.001
DHS1P	0.978	0.694	1.095	0.413	0.293	1.062	=0.001
**Sum**							
Cers	7.494	5.950	9.398	6.154	5.462	7.635	=0.053
DHCers	0.329	0.287	0.375	0.293	0.244	0.400	=0.231
SMs	525.966	470.127	556.727	292.808	233.854	419.931	<0.001
HexCers	17.816	16.181	22.274	18.401	16.562	19.909	=0.963
LacCers	10.725	8.700	11.908	12.993	9.491	14.612	=0.017
GM3s	5.687	4.763	6.953	6.775	3.540	10.826	=0.669

Note: Data, expressed as median and interquartile range [25th and 75th], were analyzed by the Mann–Whitney Rank Sum Test. See text for abbreviations.

## Data Availability

The datasets used and/or analyzed in the present study will be uploaded on www.zenodo.org and available from the corresponding author upon a reasonable request.

## Supplementary Materials

The following supporting information can be downloaded at: https://www.mdpi.com/article/10.3390/jcm14186482/s1, Tables S1–S4: “Correlations”.

## Abbreviations

ACTH, adrenocorticotropic hormone; AN, anorexia nervosa; BMI, body mass index; BW, body weight; Cer, cremide; CerS, ceramide synthase; CoA, coenzyme A; CRH, corticotropin-releasing hormone; CRP, C reactive protein; DHCer, dihydroceramide; DHS1P, dihydrosphingosine-1-phosphate; DHsph, dihydrosphingosine; FFM, free fat mass; FM, fat mass; GH, growth hormone; GM3, monosialodihexosylganglioside; GnRH, gonadotropin-releasing hormone; HBA1c, glycated haemoglobin; HDL-C, high-density lipoprotein cholesterol; HexCer, hexosyl-ceramide; HOMA-IR, homeostatic model assessment for insulin resistance; IGF-1, insulin-like growth factor 1; LacCer, lactosyl-ceramide; LDL-C, low-density lipoprotein cholesterol; NWH, normal-weight healthy: REE, resting energy expenditure; SM, sphingomyelin; S1P, sphingosine-1-phosphate; sph, sphingosine; T-C, total cholesterol; TG, triglycerides; TNF-α, tumor necrosis factor α; TSH, thyroid-stimulating hormone.

## References

[1] Bulik C.M., Bulik C.M., Carroll I.M., Mehler P. (2021). Reframing Anorexia Nervosa as a Metabo-Psychiatric Disorder. Trends Endocrinol. Metab..

[2] Watson H.J., Yilmaz Z., Thornton L.M., Hübel C., Coleman J.R.I., Gaspar H., Bryois J., Hinney A., Leppä V.M., Mattheisen M. (2019). Genome-Wide Association Study Identifies Eight Risk Loci and Implicates Metabo-Psychiatric Origins for Anorexia Nervosa. Nat. Genet..

[3] Chesney E., Goodwin G.M., Fazel S. (2014). Risks of All-Cause and Suicide Mortality in Mental Disorders: A Meta-Review. World Psychiatry.

[4] Edition F. (2013). Diagnostic and Statistical Manual of Mental Disorders, Fifth Edition. Am. Psychiatr. Assoc..

[5] Auger N., Potter B.J., Ukah U.V., Low N., Israel M., Israel M., Steiger H., Steiger H., Healy-Profitós J., Paradis G. (2021). Anorexia Nervosa and the Long-Term Risk of Mortality in Women. World Psychiatry.

[6] Van Eeden A.E., van Hoeken D., Hoek H.W. (2021). Incidence, prevalence and mortality of anorexia nervosa and bulimia nervosa. Curr. Opin. Psychiatry.

[7] Eddy K.T., Tabri N., Thomas J.J., Murray H.B., Keshaviah A., Hastings E., Edkins K., Krishna M., Herzog D.B., Keel P.K. (2017). Recovery from Anorexia Nervosa and Bulimia Nervosa at 22-Year Follow-Up. J. Clin. Psychiatry.

[8] Herpertz-Dahlmann B., Dempfle A., Egberts K., Kappel V., Konrad K., Vloet J.A., Bühren K. (2018). Outcome of Childhood Anorexia Nervosa-The Results of a Five- to Ten-Year Follow-up Study. Int. J. Eat. Disord..

[9] Mairhofer D., Zeiler M., Philipp J., Truttmann S., Wittek T., Skala K., Mitterer M., Schöfbeck G., Laczkovics C., Schwarzenberg J. (2021). Short-Term Outcome of Inpatient Treatment for Adolescents with Anorexia Nervosa Using DSM-5 Remission Criteria. J. Clin. Med..

[10] Silén Y., Sipilä P.N., Raevuori A., Raevuori A., Mustelin L., Marttunen M., Kaprio J., Keski-Rahkonen A. (2021). Detection, Treatment, and Course of Eating Disorders in Finland: A Population-Based Study of Adolescent and Young Adult Females and Males. Eur. Eat. Disord. Rev..

[11] Duncan L.E., Yilmaz Z., Gaspar H., Walters R.K., Goldstein J., Anttila V., Bulik-Sullivan B., Ripke S., Thornton L.M., Hinney A. (2017). Significant Locus and Metabolic Genetic Correlations Revealed in Genome-Wide Association Study of Anorexia Nervosa. Am. J. Psychiatry.

[12] Hussain A.A., Hübel C., Hübel C., Hindborg M., Lindkvist E.B., Kastrup A.M., Yilmaz Z., Støving R.K., Bulik C.M., Bulik C.M. (2019). Increased Lipid and Lipoprotein Concentrations in Anorexia Nervosa: A Systematic Review and Meta-Analysis. Int. J. Eat. Disord..

[13] Soemedi H. (2022). The Foundations and Development of Lipidomics. J. Lipid Res..

[14] Shevchenko A., Simons K. (2010). Lipidomics: Coming to Grips with Lipid Diversity. Nat. Rev. Mol. Cell Biol..

[15] van Meer G. (2005). Cellular lipidomics. EMBO J..

[16] Hannun Y.A., Obeid L.M., Obeid L.M. (2008). Principles of Bioactive Lipid Signalling: Lessons from Sphingolipids. Nat. Rev. Mol. Cell Biol..

[17] Wymann M.P., Schneiter R. (2008). Lipid Signalling in Disease. Nat. Rev. Mol. Cell Biol..

[18] van Kruining D., Luo Q., van Echten-Deckert G., Mielke M.M., Bowman A.P., Ellis S.R., Gil Oliveira T., Martinez-Martinez P. (2020). Sphingolipids as Prognostic Biomarkers of Neurodegeneration, Neuroinflammation, and Psychiatric Diseases and Their Emerging Role in Lipidomic Investigation Methods. Adv. Drug Deliv. Rev..

[19] Favaro A., Caregaro L., Di Pascoli L., Brambilla F., Santonastaso P. (2004). Total Serum Cholesterol and Suicidality in Anorexia Nervosa. Psychosom. Med..

[20] Tam F.I., Gerl M.J., Klose C., Surma M.A., King J.A., Seidel M., Weidner K., Roessner V., Simons K., Ehrlich S. (2021). Adverse Effects of Refeeding on the Plasma Lipidome in Young Individuals with Anorexia Nervosa. J. Am. Acad. Child Adolesc. Psychiatry.

[21] Hussain A.A., Bilgin M., Carlsson J., Foged M.M., Mortensen E.L., Bulik C.M., Støving R.K., Sjögren J.M. (2023). Elevated Lipid Class Concentrations in Females with Anorexia Nervosa before and after Intensive Weight Restoration Treatment—A Lipidomics Study. Int. J. Eat. Disord..

[22] Capuano E.I., Ruocco A., Scazzocchio B., Zanchi G., Lombardo C., Silenzi A., Ortona E., Varì R. (2025). Gender differences in eating disorders. Front. Nutr..

[23] First M.B., Skodol A.E., Bender D.S., Oldham J.M. (2016). User’s Guide for the Structured Clinical Interview for the DSM-5Â^®^ Alternative Model for Personality Disorders (SCID-5-AMPD).

[24] Brusa F., Scarpina F., Bastoni I., Villa V., Castelnuovo G., Apicella E., Savino S., Mendolicchio L. (2023). Short-Term Effects of a Multidisciplinary Inpatient Intensive Rehabilitation Treatment on Body Image in Anorexia Nervosa. J. Eat. Disord..

[25] Alleva J.M., Sheeran P., Webb T.L., Martijn C., Miles E. (2015). A Meta-Analytic Review of Stand-Alone Interventions to Improve Body Image. PLoS ONE.

[26] Casper R.C., Voderholzer U., Naab S., Schlegl S. (2020). Increased Urge for Movement, Physical and Mental Restlessness, Fundamental Symptoms of Restricting Anorexia Nervosa?. Brain Behav..

[27] Rizk M., Mattar L., Kern L., Berthoz S., Duclos J., Viltart O., Godart N. (2020). Physical Activity in Eating Disorders: A Systematic Review. Nutrients.

[28] Pieters G., Vansteelandt K., Claes L., Probst M., Van Mechelen I., Vandereycken W. (2006). The Usefulness of Experience Sampling in Understanding the Urge to Move in Anorexia Nervosa. Acta Neuropsychiatr..

[29] Madden S., Hay P., Touyz S. (2015). Systematic Review of Evidence for Different Treatment Settings in Anorexia Nervosa. World J. Psychiatry.

[30] Zipfel S., Giel K.E., Giel K.E., Bulik C.M., Bulik C.M., Hay P., Schmidt U. (2015). Anorexia Nervosa: Aetiology, Assessment, and Treatment. Lancet Psychiatry.

[31] McClave S.A., Lowen C.C., Kleber M.J., McConnell J.W., Jung L.Y., Goldsmith L.J. (2003). Clinical use of the respiratory quotient obtained from indirect calorimetry. J. Parenter. Enter. Nutr..

[32] Weir J.B.D.B. (1949). New methods for calculating metabolic rate with special reference to protein metabolism. J. Physiol..

[33] Merrill A.H., Sullards M.C., Allegood J.C., Kelly S., Wang E. (2005). Sphingolipidomics: High-Throughput, Structure-Specific, and Quantitative Analysis of Sphingolipids by Liquid Chromatography Tandem Mass Spectrometry. Methods.

[34] Platania C.B.M., Dei Cas M., Cianciolo S., Fidilio A., Lazzara F., Paroni R., Pignatello R., Strettoi E., Ghidoni R., Drago F. (2019). Novel Ophthalmic Formulation of Myriocin: Implications in Retinitis Pigmentosa. Drug Deliv..

[35] Morano C., Zulueta A., Caretti A., Roda G., Paroni R., Dei Cas M. (2022). An Update on Sphingolipidomics: Is Something Still Missing? Some Considerations on the Analysis of Complex Sphingolipids and Free-Sphingoid Bases in Plasma and Red Blood Cells. Metabolites.

[36] Boini K.M., Xia M., Koka S., Gehr T.W.B., Li P.-L. (2017). Sphingolipids in Obesity and Related Complications. Front. Biosci..

[37] Straczkowski M., Kowalska I. (2008). The Role of Skeletal Muscle Sphingolipids in the Development of Insulin Resistance. Rev. Diabet. Stud. RDS.

[38] Kang S.C., Kim B.-R., Lee S.-Y., Park T.S. (2013). Sphingolipid Metabolism and Obesity-Induced Inflammation. Front. Endocrinol..

[39] Xiao Y., Liu D., Cline M.A., Gilbert E.R. (2020). Chronic Stress and Adipose Tissue in the Anorexic State: Endocrine and Epigenetic Mechanisms. Adipocyte.

[40] El Ghoch M., Calugi S., Lamburghini S., Dalle Grave R. (2014). Anorexia Nervosa and Body Fat Distribution: A Systematic Review. Nutrients.

[41] Pasanisi F., Pace L., Fonti R., Marra M., Sgambati D., De Caprio C., De Filippo E., Vaccaro A., Salvatore M., Contaldo F. (2013). Evidence of Brown Fat Activity in Constitutional Leanness. J. Clin. Endocrinol. Metab..

[42] Li Y., Talbot C.L., Chaurasia B., Chaurasia B. (2020). Ceramides in Adipose Tissue. Front. Endocrinol..

[43] Bikman B.T., Summers S.A. (2011). Ceramides as Modulators of Cellular and Whole-Body Metabolism. J. Clin. Investig..

[44] Bruni P., Donati C. (2008). Pleiotropic Effects of Sphingolipids in Skeletal Muscle. Cell. Mol. Life Sci..

[45] Rosa-Caldwell M.E., Eddy K.T., Rutkove S.B., Breithaupt L. (2022). Anorexia Nervosa and Muscle Health: A Systematic Review of Our Current Understanding and Future Recommendations for Study. Int. J. Eat. Disord..

[46] Kojima M., Hosoda H., Date Y., Nakazato M., Matsuo H., Kangawa K. (1999). Ghrelin Is a Growth-Hormone-Releasing Acylated Peptide from Stomach. Nature.

[47] Louveau I., Gondret F. (2004). Regulation of Development and Metabolism of Adipose Tissue by Growth Hormone and the Insulin-like Growth Factor System. Domest. Anim. Endocrinol..

[48] Sovetkina A., Nadir R., Fung J.N.M., Nadjarpour A., Beddoe B. (2020). The Physiological Role of Ghrelin in the Regulation of Energy and Glucose Homeostasis. Cureus.

[49] Wang Y., Nishi M., Doi A., Shono T., Furukawa Y., Shimada T., Furuta H., Sasaki H., Nanjo K. (2010). Ghrelin Inhibits Insulin Secretion through the AMPK–UCP2 Pathway in β Cells. FEBS Lett..

[50] Rigamonti A.E., Pincelli A.I., Corra B., Viarengo R., Bonomo S.M., Galimberti D., Scacchi M., Scarpini E., Cavagnini F., Müller E.E. (2002). COMMUNICATION: Plasma Ghrelin Concentrations in Elderly Subjects: Comparison with Anorexic and Obese Patients. J. Endocrinol..

[51] Prioletta A., Muscogiuri G., Sorice G.P., Lassandro A.P., Mezza T., Policola C., Salomone E., Cipolla C., Della Casa S., Pontecorvi A. (2011). In Anorexia Nervosa, Even a Small Increase in Abdominal Fat Is Responsible for the Appearance of Insulin Resistance. Clin. Endocrinol..

[52] Hebebrand J., Blum W.F., Blum W.F., Barth N., Coners H., Englaro P., Juul A., Ziegler A., Warnke A., Rascher W. (1997). Leptin Levels in Patients with Anorexia Nervosa Are Reduced in the Acute Stage and Elevated upon Short-Term Weight Restoration. Mol. Psychiatry.

[53] Pereira S., Cline D.L., Glavas M.M., Covey S.D., Kieffer T.J. (2021). Tissue-Specific Effects of Leptin on Glucose and Lipid Metabolism. Endocr. Rev..

[54] Skorupskaite K., George J.T., Anderson R.A. (2014). The Kisspeptin-GnRH Pathway in Human Reproductive Health and Disease. Hum. Reprod. Update.

[55] Schorr M., Miller K.K. (2017). The Endocrine Manifestations of Anorexia Nervosa: Mechanisms and Management. Nat. Rev. Endocrinol..

[56] Li J., Xie L.M., Song J.L., Yau L.F., Mi J.N., Zhang C.R., Wu W.T., Lai M.H., Jiang Z.H., Wang J.R. (2019). Alterations of Sphingolipid Metabolism in Different Types of Polycystic Ovary Syndrome. Sci. Rep..

[57] Lucki N.C., Sewer M.B. (2010). The interplay between bioactive sphingolipids and steroid hormones. Steroids.

[58] Torretta E., Barbacini P., Al-Daghri N.M., Gelfi C. (2019). Sphingolipids in Obesity and Correlated Co-Morbidities: The Contribution of Gender, Age and Environment. Int. J. Mol. Sci..

[59] Suemaru S., Hashimoto K., Hattori T., Inoue H., Kageyama J., Ota Z. (1986). Starvation-Induced Changes in Rat Brain Corticotropin-Releasing Factor (CRF) and Pituitary-Adrenocortical Response. Life Sci..

[60] Björntorp P. (1997). Hormonal Control of Regional Fat Distribution. Hum. Reprod..

[61] Rubello D., Sonino N., Casara D., Girelli M.E., Busnardo B., Boscaro M. (1992). Acute and Chronic Effects of High Glucocorticoid Levels on Hypothalamic-Pituitary-Thyroid Axis in Man. J. Endocrinol. Investig..

[62] Kluge M., Riedl S., Uhr M., Schmidt D., Zhang X., Yassouridis A., Steiger A. (2010). Ghrelin Affects the Hypothalamus-Pituitary-Thyroid Axis in Humans by Increasing Free Thyroxine and Decreasing TSH in Plasma. Eur. J. Endocrinol..

[63] Xu C., Mutwalli H., Haslam R., Keeler J.L., Treasure J., Himmerich H. (2024). C-Reactive Protein (CRP) Levels in People with Eating Disorders: A Systematic Review and Meta-Analysis. J. Psychiatr. Res..

[64] Nakai Y., Hamagaki S., Takagi R., Taniguchi A., Kurimoto F. (2001). Plasma Concentrations of Tumor Necrosis Factor-α (TNF-α) and Soluble TNF Receptors in Patients with Bulimia Nervosa. Clin. Endocrinol..

[65] Lee M.Y., Lee S., Bae Y.-S. (2023). Functional Roles of Sphingolipids in Immunity and Their Implication in Disease. Exp. Mol. Med..

[66] Casper R.C. (1996). Carbohydrate Metabolism and Its Regulatory Hormones in Anorexia Nervosa. Psychiatry Res.-Neuroimaging.

[67] Borodzicz-Jazdzyk S., Jażdżyk P., Łysik W., Cudnoch-Jędrzejewska A., Czarzasta K. (2022). Sphingolipid Metabolism and Signaling in Cardiovascular Diseases. Front. Cardiovasc. Med..

[68] Mullen T.D., Hannun Y.A., Obeid L.M., Obeid L.M. (2012). Ceramide Synthases at the Centre of Sphingolipid Metabolism and Biology. Biochem. J..

[69] Wattenberg B.W. (2018). The Long and the Short of Ceramides. J. Biol. Chem..

[70] Shimizu M., Kawai K., Yamashita M., Shoji M., Takakura S., Hata T., Nakashima M., Tatsushima K., Tanaka K., Sudo N. (2020). Very Long Chain Fatty Acids Are an Important Marker of Nutritional Status in Patients with Anorexia Nervosa: A Case Control Study. Biopsychosoc. Med..

