# Supplementary material for: Evaluation of the Sphingolipidomic Profile in Women with Anorexia Nervosa: Relationships with Parameters Related to Body Composition, Cardiovascular Function, Glucometabolic Homeostasis, and Lipoprotein Metabolism
Source: J Clin Med. 2025 Sep 15;14(18):6482. doi: 10.3390/jcm14186482 (PMC12470723; doi:10.3390/jcm14186482)
Supplement: Supplementary file 1 [file jcm-14-06482-s001.zip › Table S1.pdf]

Table S1. Correlations of single/total sphingolipids with age and parameters related to body composition.

Sphingolipid	Age	BMI	FFM (kg)	FFM (%)	FM (kg)	FM (%)	REE
Cer 14:0	-0.014	-0.154	-0.141	0.177	-0.187	-0.165	-0.259
	0.919	0.246	0.293	0.186	0.164	0.218	0.050
Cer 16:0	0.071	-0.148	-0.172	0.132	-0.139	-0.130	-0.115
	0.598	0.266	0.200	0.327	0.301	0.335	0.387
Cer 18:1	0.127	0.064	0.091	0.010	0.022	0.006	0.107
	0.341	0.630	0.498	0.938	0.870	0.967	0.422
Cer 18:0	-0.065	-0.022	-0.094	-0.088	0.029	0.083	0.062
	0.626	0.868	0.484	0.515	0.829	0.539	0.645
Cer 20:0	-0.193	-0.463	-0.447	0.308	-0.413	-0.316	-0.292
	0.146	0.000	0.001	0.020	0.001	0.017	0.027
Cer 22:0	0.009	0.300	0.220	-0.319	0.311	0.324	0.319
	0.947	0.022	0.101	0.016	0.019	0.014	0.015
Cer 24:1	-0.302	-0.714	-0.625	0.622	-0.711	-0.628	-0.534
	0.021	0.000	0.000	0.000	0.000	0.000	0.000
Cer 24:0	-0.062	-0.021	-0.102	-0.026	-0.030	0.024	0.050
	0.641	0.874	0.447	0.844	0.824	0.858	0.710
DHCer 16:0	0.209	0.137	0.178	-0.083	0.132	0.100	0.117
	0.116	0.304	0.184	0.538	0.325	0.460	0.380
DHCer 18:1	0.249	0.619	0.568	-0.473	0.575	0.485	0.429
	0.060	0.000	0.000	0.000	0.000	0.000	0.001
DHCer 18:0	-0.028	0.111	-0.017	-0.160	0.123	0.145	0.043
	0.850	0.457	0.910	0.288	0.414	0.334	0.771
DHCer 24:1	-0.270	-0.418	-0.317	0.316	-0.344	-0.321	-0.309
	0.040	0.001	0.016	0.017	0.009	0.015	0.019
DHCer 24:0	0.058	0.338	0.288	-0.261	0.321	0.269	0.236
	0.667	0.010	0.030	0.050	0.015	0.043	0.074
SM 16:0	-0.195	-0.631	-0.528	0.563	-0.638	-0.570	-0.480
	0.142	0.000	0.000	0.000	0.000	0.000	0.000
SM 18:0	-0.077	-0.081	-0.136	0.033	-0.083	-0.040	-0.008
	0.567	0.545	0.311	0.807	0.539	0.766	0.951
SM 18:1	-0.022	-0.018	-0.094	-0.022	-0.042	0.016	0.050
	0.870	0.895	0.484	0.869	0.757	0.905	0.709
SM 24:0	-0.176	-0.445	-0.416	0.384	-0.454	-0.396	-0.302
	0.185	0.001	0.001	0.003	0.000	0.002	0.021
SM 24:1	-0.306	-0.732	-0.632	0.616	-0.699	-0.627	-0.538
	0.020	0.000	0.000	0.000	0.000	0.000	0.000
Total Cer	-0.120	-0.182	-0.225	0.113	-0.178	-0.115	-0.081
	0.367	0.172	0.093	0.402	0.185	0.395	0.542
Total DHCer	-0.072	0.025	0.036	-0.001	0.027	0.003	-0.025
	0.592	0.853	0.788	0.995	0.839	0.984	0.851
Total SM	-0.241	-0.604	-0.537	0.514	-0.596	-0.524	-0.436
	0.068	0.000	0.000	0.000	0.000	0.000	0.001
HexCer 16:0	0.141	0.048	0.044	-0.105	0.126	0.107	0.013
	0.290	0.717	0.745	0.437	0.349	0.428	0.920

HexCer 18:0	0.151	0.020	0.049	-0.040	0.048	0.047	-0.047
	0.258	0.880	0.718	0.765	0.722	0.730	0.728
HexCer 18:1	0.034	0.565	0.399	-0.447	0.490	0.461	0.404
	0.802	0.000	0.002	0.001	0.000	0.000	0.002
HexCer 20:0	-0.004	-0.041	-0.054	-0.019	0.003	0.016	0.063
	0.973	0.759	0.687	0.889	0.984	0.908	0.640
HexCer 22:0	-0.009	0.147	0.050	-0.320	0.277	0.327	0.235
	0.948	0.269	0.710	0.015	0.037	0.013	0.075
HexCer 24:0	0.046	-0.052	-0.179	-0.128	0.062	0.123	-0.025
	0.730	0.700	0.181	0.342	0.645	0.360	0.854
HexCer 24:1	-0.245	-0.623	-0.573	0.396	-0.489	-0.402	-0.434
	0.064	0.000	0.000	0.002	0.000	0.002	0.001
LacCer 16:0	-0.042	0.202	0.227	-0.184	0.234	0.187	0.184
	0.756	0.128	0.089	0.170	0.079	0.164	0.165
LacCer 18:0	0.008	0.374	0.374	-0.329	0.382	0.334	0.309
	0.952	0.004	0.004	0.013	0.004	0.011	0.018
LacCer 18:1	-0.120	-0.244	-0.241	0.132	-0.144	-0.137	-0.190
	0.367	0.065	0.071	0.326	0.286	0.310	0.153
LacCer 20:0	-0.043	-0.075	-0.089	-0.018	0.002	0.018	-0.077
	0.750	0.573	0.511	0.893	0.989	0.895	0.563
LacCer 22:0	-0.129	-0.034	-0.117	-0.086	0.040	0.084	0.042
	0.332	0.800	0.383	0.526	0.765	0.532	0.754
LacCer 24:0	-0.135	-0.281	-0.360	0.193	-0.251	-0.198	-0.244
	0.313	0.033	0.006	0.149	0.060	0.140	0.065
LacCer 24:1	-0.151	-0.397	-0.403	0.259	-0.311	-0.258	-0.279
	0.256	0.002	0.002	0.052	0.019	0.053	0.034
GM3 16:0	-0.106	-0.259	-0.154	0.243	-0.219	-0.239	-0.216
	0.427	0.050	0.252	0.069	0.102	0.073	0.103
GM3 18:0	-0.220	-0.115	-0.123	0.083	-0.100	-0.087	-0.046
	0.098	0.389	0.360	0.536	0.458	0.519	0.732
GM3 18:1	0.267	0.583	0.545	-0.415	0.533	0.427	0.387
	0.043	0.000	0.000	0.001	0.000	0.001	0.003
GM3 20:0	-0.124	-0.564	-0.454	0.473	-0.492	-0.474	-0.417
	0.354	0.000	0.000	0.000	0.000	0.000	0.001
GM3 22:0	0.091	0.183	0.179	-0.137	0.177	0.154	0.105
	0.496	0.169	0.181	0.310	0.187	0.251	0.433
GM3 24:0	-0.057	-0.295	-0.282	0.273	-0.316	-0.267	-0.240
	0.669	0.025	0.034	0.040	0.017	0.044	0.069
GM3 24:1	-0.126	-0.502	-0.348	0.495	-0.503	-0.488	-0.410
	0.343	0.000	0.008	0.000	0.000	0.000	0.001
Total HexCer	0.095	0.061	0.054	-0.181	0.162	0.190	0.110
	0.563	0.709	0.747	0.276	0.330	0.251	0.502
Total LacCer	0.048	0.294	0.290	-0.301	0.346	0.312	0.239
	0.719	0.025	0.029	0.023	0.009	0.018	0.070
Total GM3	0.053	0.053	0.115	0.005	0.063	0.008	-0.016
	0.694	0.691	0.395	0.971	0.640	0.952	0.907
Sph	0.170	0.040	-0.019	0.057	-0.051	-0.049	-0.045
	0.202	0.766	0.889	0.671	0.706	0.719	0.737

S1P	-0.117	-0.161	-0.215	0.026	-0.086	-0.039	-0.016
	0.380	0.227	0.108	0.848	0.521	0.770	0.907
DhSph	0.279	0.581	0.543	-0.438	0.543	0.451	0.396
	0.034	0.000	0.000	0.001	0.000	0.000	0.002
DhS1P	-0.121	-0.359	-0.361	0.176	-0.262	-0.188	-0.232
	0.363	0.006	0.006	0.190	0.049	0.161	0.080

Note: Each cell contains the correlation coefficient (above) and p value (below). The correlation coefficient was calculated based on Spearman’s correlation.
