# Supplementary material for: Evaluation of the Sphingolipidomic Profile in Women with Anorexia Nervosa: Relationships with Parameters Related to Body Composition, Cardiovascular Function, Glucometabolic Homeostasis, and Lipoprotein Metabolism
Source: J Clin Med. 2025 Sep 15;14(18):6482. doi: 10.3390/jcm14186482 (PMC12470723; doi:10.3390/jcm14186482)
Supplement: Supplementary file 1 [file jcm-14-06482-s001.zip › Table S2.pdf]

Table S2. Correlations of single/total sphingolipids with parameters related to cardiovascular function.

Sphingolipid	SBP	DBP	HR
Cer 14:0	-0.144	-0.011	0.045
	0.278	0.936	0.739
Cer 16:0	0.108	0.240	-0.031
	0.417	0.070	0.816
Cer 18:1	0.156	0.276	0.017
	0.242	0.036	0.898
Cer 18:0	0.140	0.358	0.173
	0.294	0.006	0.194
Cer 20:0	-0.209	0.092	0.205
	0.116	0.493	0.122
Cer 22:0	0.383	0.362	0.291
	0.003	0.005	0.027
Cer 24:1	-0.497	-0.152	0.191
	0.000	0.254	0.151
Cer 24:0	0.136	0.198	0.195
	0.308	0.136	0.142
DHCer 16:0	0.293	0.371	-0.003
	0.026	0.004	0.980
DHCer 18:1	0.424	0.236	0.037
	0.001	0.074	0.784
DHCer 18:0	0.158	0.166	0.134
	0.287	0.264	0.368
DHCer 24:1	-0.318	-0.092	0.130
	0.015	0.492	0.328
DHCer 24:0	0.316	0.274	0.019
	0.016	0.037	0.886
SM 16:0	-0.393	-0.101	0.080
	0.002	0.451	0.550
SM 18:0	0.045	0.237	0.137
	0.738	0.073	0.303
SM 18:1	0.114	0.286	0.074
	0.392	0.030	0.580
SM 24:0	-0.231	-0.040	0.027
	0.081	0.764	0.840
SM 24:1	-0.509	-0.190	0.095
	0.000	0.154	0.479
Total Cer	0.018	0.160	0.212
	0.895	0.230	0.109
Total DHCer	0.066	0.156	0.082
	0.624	0.241	0.538
Total SM	-0.375	-0.074	0.109
	0.004	0.580	0.415
HexCer 16:0	0.256	0.441	-0.034
	0.052	0.001	0.797
HexCer 18:0	0.175	0.388	0.038

	0.189	0.003	0.774
HexCer 18:1	0.402	0.210	0.005
	0.002	0.113	0.969
HexCer 20:0	0.009	0.285	0.045
	0.945	0.030	0.737
HexCer 22:0	0.237	0.265	-0.089
	0.074	0.045	0.506
HexCer 24:0	0.023	0.107	-0.090
	0.864	0.423	0.501
HexCer 24:1	-0.424	-0.051	0.033
	0.001	0.705	0.807
LacCer 16:0	0.305	0.447	0.261
	0.020	0.000	0.048
LacCer 18:0	0.316	0.444	0.179
	0.016	0.001	0.177
LacCer 18:1	0.027	0.217	0.150
	0.839	0.102	0.261
LacCer 20:0	0.001	0.266	0.189
	0.992	0.044	0.156
LacCer 22:0	-0.004	0.156	0.225
	0.973	0.241	0.089
LacCer 24:0	-0.208	0.025	0.162
	0.117	0.854	0.222
LacCer 24:1	-0.205	0.092	0.224
	0.122	0.490	0.091
GM3 16:0	-0.144	0.183	0.097
	0.279	0.168	0.466
GM3 18:0	-0.189	0.083	0.245
	0.155	0.533	0.064
GM3 18:1	0.361	0.215	0.004
	0.005	0.105	0.974
GM3 20:0	-0.308	0.018	0.138
	0.019	0.895	0.301
GM3 22:0	0.238	0.351	0.203
	0.072	0.007	0.125
GM3 24:0	-0.212	0.041	0.276
	0.110	0.757	0.036
GM3 24:1	-0.358	0.018	0.178
	0.006	0.894	0.181
Total HexCer	0.095	0.330	-0.032
	0.563	0.040	0.846
Total LacCer	0.382	0.440	0.216
	0.003	0.001	0.103
Total GM3	0.069	0.227	0.122
	0.607	0.087	0.361
Total Sph	-0.006	0.081	0.045
	0.965	0.542	0.735
S1P	0.125	0.230	-0.095

	0.348	0.082	0.476
DhSph	0.406	0.208	-0.023
	0.002	0.116	0.866
DhS1P	-0.067	0.097	0.034
	0.614	0.467	0.802

Note: Each cell contains the correlation coefficient (above) and p value (below). The correlation coefficient was calculated based on Spearman’s correlation.
