# Supplementary material for: Evaluation of the Sphingolipidomic Profile in Women with Anorexia Nervosa: Relationships with Parameters Related to Body Composition, Cardiovascular Function, Glucometabolic Homeostasis, and Lipoprotein Metabolism
Source: J Clin Med. 2025 Sep 15;14(18):6482. doi: 10.3390/jcm14186482 (PMC12470723; doi:10.3390/jcm14186482)
Supplement: Supplementary file 1 [file jcm-14-06482-s001.zip › Table S3.pdf]

Table S3. Correlations of single/total sphingolipids with parameters related to glucometabolic homeostasis.

**Sphingolipid**	**Glucose**	**Insulin**	**HOMA-IR**	**HbA1c**
Cer 14:0	0.018	-0.092	-0.080	0.024
	0.892	0.490	0.549	0.857
Cer 16:0	0.011	0.037	0.029	0.278
	0.935	0.784	0.828	0.035
Cer 18:1	0.101	0.000	-0.011	0.282
	0.447	0.999	0.933	0.032
Cer 18:0	0.153	0.133	0.130	0.483
	0.250	0.318	0.329	0.000
Cer 20:0	-0.173	-0.172	-0.195	0.196
	0.193	0.197	0.142	0.140
Cer 22:0	0.290	0.127	0.167	0.403
	0.028	0.341	0.209	0.002
Cer 24:1	-0.420	-0.351	-0.387	-0.076
	0.001	0.007	0.003	0.569
Cer 24:0	0.040	-0.060	-0.039	0.291
	0.767	0.652	0.770	0.027
DHCer 16:0	0.067	0.055	0.063	0.398
	0.615	0.679	0.639	0.002
DHCer 18:1	0.377	0.326	0.353	0.135
	0.004	0.013	0.007	0.311
DHCer 18:0	0.313	0.352	0.368	0.147
	0.032	0.015	0.011	0.322
DHCer 24:1	-0.189	-0.054	-0.080	0.038
	0.155	0.687	0.551	0.774
DHCer 24:0	0.262	0.174	0.206	0.419
	0.047	0.190	0.120	0.001
SM 16:0	-0.452	-0.341	-0.381	-0.020
	0.000	0.009	0.003	0.882
SM 18:0	-0.006	0.070	0.049	0.415
	0.961	0.600	0.711	0.001
SM 18:1	-0.025	-0.004	-0.020	0.438
	0.853	0.979	0.879	0.001
SM 24:0	-0.316	-0.152	-0.186	0.011
	0.016	0.254	0.162	0.932
SM 24:1	-0.502	-0.307	-0.358	-0.126
	0.000	0.020	0.006	0.343
Total Cer	-0.036	-0.120	-0.107	0.266
	0.785	0.369	0.424	0.043
Total DHCer	0.045	0.086	0.087	0.267
	0.738	0.520	0.516	0.043
Total SM	-0.437	-0.266	-0.312	-0.008
	0.001	0.044	0.017	0.955
HexCer 16:0	0.167	0.259	0.249	0.303
	0.210	0.050	0.059	0.021

HexCer 18:0	0.159	0.092	0.091	0.445
	0.232	0.490	0.495	0.001
HexCer 18:1	0.320	0.338	0.356	0.136
	0.015	0.010	0.006	0.306
HexCer 20:0	-0.027	0.117	0.082	0.112
	0.843	0.382	0.537	0.400
HexCer 22:0	0.129	0.203	0.211	-0.004
	0.332	0.127	0.112	0.976
HexCer 24:0	0.040	0.106	0.117	-0.047
	0.767	0.429	0.379	0.725
HexCer 24:1	-0.285	-0.177	-0.207	-0.084
	0.030	0.183	0.118	0.528
LacCer 16:0	0.245	0.333	0.336	0.231
	0.064	0.011	0.010	0.081
LacCer 18:0	0.303	0.251	0.267	0.352
	0.021	0.058	0.043	0.007
LacCer 18:1	-0.160	0.107	0.068	0.034
	0.230	0.421	0.611	0.801
LacCer 20:0	0.057	0.113	0.104	0.150
	0.672	0.399	0.436	0.260
LacCer 22:0	-0.014	0.112	0.110	-0.013
	0.917	0.401	0.410	0.925
LacCer 24:0	-0.274	-0.035	-0.064	-0.078
	0.038	0.794	0.631	0.562
LacCer 24:1	-0.268	-0.002	-0.038	-0.122
	0.042	0.986	0.776	0.360
GM3 16:0	-0.154	0.002	-0.027	0.072
	0.247	0.990	0.840	0.589
GM3 18:0	-0.126	-0.027	-0.057	0.242
	0.346	0.840	0.670	0.067
GM3 18:1	0.357	0.359	0.383	0.106
	0.006	0.006	0.003	0.427
GM3 20:0	-0.312	-0.241	-0.274	0.129
	0.018	0.068	0.038	0.333
GM3 22:0	0.080	0.093	0.093	0.098
	0.551	0.487	0.487	0.461
GM3 24:0	-0.279	-0.169	-0.191	-0.079
	0.034	0.204	0.151	0.556
GM3 24:1	-0.393	-0.261	-0.317	-0.033
	0.002	0.048	0.015	0.804
Total HexCer	0.041	0.033	0.040	-0.021
	0.803	0.839	0.805	0.897
Total LacCer	0.324	0.309	0.318	0.162
	0.013	0.019	0.015	0.224
Total GM3	0.019	0.060	0.050	0.103
	0.888	0.656	0.711	0.441
Sph	0.100	-0.015	-0.004	-0.081
	0.454	0.909	0.976	0.546

S1P	0.067	0.047	0.065	0.116
	0.618	0.725	0.628	0.383
DhSph	0.396	0.285	0.322	0.126
	0.002	0.030	0.014	0.344
DhS1P	-0.030	-0.055	-0.054	0.033
	0.822	0.683	0.686	0.805

Note: Each cell contains the correlation coefficient (above) and p value (below). The correlation coefficient was calculated based on Spearman's correlation.
