# Supplementary material for: Evaluation of the Sphingolipidomic Profile in Women with Anorexia Nervosa: Relationships with Parameters Related to Body Composition, Cardiovascular Function, Glucometabolic Homeostasis, and Lipoprotein Metabolism
Source: J Clin Med. 2025 Sep 15;14(18):6482. doi: 10.3390/jcm14186482 (PMC12470723; doi:10.3390/jcm14186482)
Supplement: Supplementary file 1 [file jcm-14-06482-s001.zip › Table S4.pdf]

Table S4. Correlations of single/total sphingolipids with parameters related to lipid metabolism and systemic inflammation.

Sphingolipid	T-C	HDL-C	LDL-C	TG	CRP
Cer 14:0	0.461	0.023	0.470	0.183	0.000
	0.000	0.866	0.000	0.169	0.999
Cer 16:0	0.494	0.153	0.420	0.272	-0.149
	0.000	0.249	0.001	0.039	0.263
Cer 18:1	0.313	0.216	0.199	-0.045	-0.133
	0.017	0.103	0.135	0.735	0.318
Cer 18:0	0.500	0.178	0.410	0.325	-0.059
	0.000	0.181	0.001	0.013	0.659
Cer 20:0	0.345	0.271	0.218	0.282	-0.430
	0.008	0.040	0.100	0.032	0.001
Cer 22:0	0.195	-0.062	0.275	0.190	0.164
	0.143	0.641	0.037	0.154	0.217
Cer 24:1	0.243	0.146	0.152	0.289	-0.542
	0.066	0.272	0.253	0.028	0.000
Cer 24:0	0.216	0.097	0.206	0.197	-0.080
	0.104	0.468	0.120	0.138	0.551
DHCer 16:0	0.241	-0.066	0.287	0.015	0.180
	0.069	0.624	0.029	0.909	0.175
DHCer 18:1	-0.034	-0.289	0.093	-0.030	0.474
	0.799	0.028	0.486	0.820	0.000
DHCer 18:0	0.353	-0.129	0.383	0.298	0.067
	0.015	0.386	0.008	0.042	0.654
DHCer 24:1	0.385	-0.087	0.369	0.324	-0.294
	0.003	0.516	0.005	0.013	0.025
DHCer 24:0	0.335	-0.277	0.448	0.145	0.315
	0.010	0.036	0.000	0.277	0.016
SM 16:0	0.335	0.264	0.209	0.171	-0.545
	0.011	0.046	0.115	0.200	0.000
SM 18:0	0.572	0.155	0.470	0.266	-0.119
	0.000	0.243	0.000	0.044	0.372
SM 18:1	0.555	0.303	0.407	0.172	-0.088
	0.000	0.021	0.002	0.195	0.512
SM 24:0	0.384	0.208	0.286	0.210	-0.308
	0.003	0.117	0.030	0.114	0.019
SM 24:1	0.298	0.193	0.181	0.243	-0.539
	0.024	0.146	0.174	0.067	0.000
Total Cer	0.284	0.125	0.251	0.283	-0.203
	0.031	0.349	0.057	0.031	0.127
Total DHCer	0.473	-0.262	0.551	0.283	0.043
	0.000	0.047	0.000	0.031	0.745
Total SM	0.403	0.238	0.272	0.252	-0.484
	0.002	0.072	0.039	0.056	0.000
HexCer 16:0	0.558	0.288	0.436	0.440	-0.035
	0.000	0.028	0.001	0.001	0.791

HexCer 18:0	0.570	0.334	0.436	0.220	-0.072
	0.000	0.011	0.001	0.096	0.592
HexCer 18:1	-0.095	-0.280	0.014	0.098	0.464
	0.478	0.034	0.919	0.463	0.000
HexCer 20:0	0.456	0.168	0.319	0.447	-0.119
	0.000	0.206	0.015	0.000	0.374
HexCer 22:0	0.235	0.154	0.189	0.274	0.003
	0.075	0.249	0.154	0.037	0.981
HexCer 24:0	0.340	0.116	0.301	0.360	-0.082
	0.009	0.386	0.022	0.006	0.539
HexCer 24:1	0.354	0.186	0.252	0.306	-0.522
	0.007	0.161	0.056	0.020	0.000
LacCer 16:0	0.416	-0.127	0.436	0.368	0.208
	0.001	0.339	0.001	0.005	0.116
LacCer 18:0	0.347	-0.128	0.379	0.147	0.285
	0.008	0.336	0.003	0.269	0.030
LacCer 18:1	0.276	0.010	0.219	0.312	-0.132
	0.036	0.940	0.099	0.017	0.321
LacCer 20:0	0.440	0.000	0.399	0.305	-0.008
	0.001	0.999	0.002	0.020	0.953
LacCer 22:0	0.257	-0.057	0.254	0.214	-0.049
	0.051	0.667	0.054	0.106	0.713
LacCer 24:0	0.271	-0.058	0.262	0.223	-0.177
	0.039	0.663	0.047	0.092	0.183
LacCer 24:1	0.278	-0.023	0.214	0.282	-0.265
	0.035	0.861	0.106	0.032	0.045
GM3 16:0	0.461	-0.060	0.437	0.333	-0.155
	0.000	0.655	0.001	0.011	0.244
GM3 18:0	0.373	-0.111	0.346	0.264	-0.062
	0.004	0.406	0.008	0.046	0.641
GM3 18:1	0.001	-0.303	0.114	0.050	0.467
	0.992	0.021	0.395	0.706	0.000
GM3 20:0	0.230	0.107	0.144	0.304	-0.333
	0.082	0.422	0.281	0.021	0.011
GM3 22:0	0.210	-0.200	0.267	0.117	0.120
	0.113	0.133	0.043	0.380	0.368
GM3 24:0	0.178	-0.126	0.200	0.064	-0.219
	0.181	0.343	0.131	0.630	0.099
GM3 24:1	0.292	0.003	0.238	0.188	-0.336
	0.026	0.981	0.072	0.156	0.010
Total HexCer	0.299	0.263	0.245	0.322	-0.265
	0.065	0.105	0.132	0.046	0.103
Total LacCer	0.263	-0.116	0.290	0.272	0.169
	0.047	0.383	0.028	0.039	0.203
Total GM3	0.243	-0.187	0.289	0.218	0.126
	0.066	0.159	0.028	0.101	0.343
Sph	0.152	0.032	0.094	0.282	0.158
	0.252	0.813	0.480	0.032	0.236

S1P	0.231<br>0.081	0.395<br>0.002	0.066<br>0.622	0.216<br>0.103	-0.128<br>0.335
DhSph	-0.075<br>0.573	-0.260<br>0.049	0.047<br>0.725	-0.075<br>0.574	0.447<br>0.000
DhS1P	0.303<br>0.021	0.390<br>0.003	0.131<br>0.324	0.161<br>0.225	-0.366<br>0.005

Note: Each cell contains the correlation coefficient (above) and p value (below). The correlation coefficient was calculated based on Spearman's correlation.
